# Valaciclovir to prevent Cytomegalovirus mediated adverse modulation of the immune system in ANCA-associated vasculitis (CANVAS): study protocol for a randomised controlled trial

**DOI:** 10.1186/s13063-016-1482-2

**Published:** 2016-07-22

**Authors:** Dimitrios Chanouzas, Lovesh Dyall, Peter Nightingale, Charles Ferro, Paul Moss, Matthew David Morgan, Lorraine Harper

**Affiliations:** School of Immunity and Infection, College of Medical and Dental Sciences, University of Birmingham, Birmingham, UK; Wolfson Computer Laboratory, Queen Elizabeth Hospital Birmingham, Birmingham, UK; School of Cancer Sciences, College of Medical and Dental Sciences, University of Birmingham, Birmingham, UK

## Abstract

**Background:**

The ANCA-associated vasculitides (AAV) are systemic autoimmune inflammatory disorders characterised by necrotising inflammation affecting small to medium-sized blood vessels. Despite improvements in survival, infection and cardiovascular disease remain leading causes of morbidity and mortality.

Considerable evidence suggests that CD4 + CD28null T-cell expansions, predominantly seen in Cytomegalovirus (CMV) seropositive individuals, are associated with systemic dysregulation of immune function leading to a heightened risk of infection and cardiovascular disease. In patients with AAV, CD4 + CD28null expansions are driven by CMV and are associated with an increased risk of infection and mortality.

The aim of this study is to explore in detail the ways in which CMV modulates the immune system and to determine whether treatment with valaciclovir blocks subclinical CMV reactivation in CMV seropositive AAV patients and ameliorates the CMV-induced adverse effects on the immune system.

**Methods/design:**

CANVAS is a single-centre prospective open-label randomised controlled proof-of-concept trial of 50 adult CMV seropositive patients with stable AAV. Participants will be randomly allocated to receive valaciclovir orally (2 g QDS or reduced according to renal function) or no additional treatment for 6 months with an additional 6-month follow-up period. The primary outcome is the proportion of patients with CMV reactivation, as assessed by measurable viral load on quantitative blood and urine CMV polymerase chain reaction. The secondary outcomes are safety, change in the proportion of CD4+ CMV-specific T-cell population (defined as CD4 + CD28null cells) and change in soluble markers of inflammation from baseline to 6 months. Further tertiary and exploratory outcomes include persistence of the effect of valaciclovir on the proportion of CD4 + CD28null cells at 6 months post completion of treatment, change in the immune phenotype of CD4+ T cells and change in blood pressure and arterial stiffness parameters from baseline to 6 months.

**Discussion:**

The results of this study will enable larger studies to be conducted to determine whether by controlling subclinical CMV reactivation, we can improve clinical endpoints such as infection and cardiovascular disease. The potential impact of this study is not limited to AAV, as CD4 + CD28null cells have been linked to adverse outcomes in other inflammatory conditions and in the context of an ageing immune system.

**Trial registration:**

ClinicalTrials.gov Identifier NCT01633476 (registered 29 June 2012).

**Electronic supplementary material:**

The online version of this article (doi:10.1186/s13063-016-1482-2) contains supplementary material, which is available to authorized users.

## Background

The anti-neutrophil cytoplasmic antibody (ANCA) associated vasculitides (AAV) are a group of rare systemic autoimmune inflammatory chronic disorders that include granulomatosis with polyangiitis (Wegener’s granulomatosis) [1], microscopic polyangiitis, renal limited vasculitis and eosinophilic granulomatosis with polyangiitis (Churg–Strauss syndrome) [2]. AAV are characterised by necrotising inflammation affecting small to medium-sized blood vessels leading to end organ damage commonly affecting the kidneys, lungs and upper airways. They range in severity from localised disease affecting the upper airways to life-threatening involvement giving rise to multi-organ failure [3].

Over the last few decades, survival has greatly improved from 20 % at 2 years to 78 % at 5 years following diagnosis [4, 5]. However, survival remains below that of the healthy population with infection, cardiovascular disease (CVD) and malignancy being the most common causes of death.

Recently the role of T cells in AAV pathophysiology and exacerbation of tissue damage has gained attention. An increased percentage of CD4 T cells that have lost expression of the co-stimulatory molecule CD28 (CD4 + CD28null cells) have been reported in AAV [6]. Loss of CD28 implies repeated antigen exposure and, in a previous study, we demonstrated that this phenotype in AAV is driven by latent Cytomegalovirus (CMV) infection [7]. An expanded CD4 + CD28null cell population in this study was associated with an increased risk of infection and increased mortality in patients with AAV. In renal transplant recipients, the presence of CD4 + CD28null cell expansions is associated with a reduced response to antigenic challenge [8] and in elderly donors this pattern is also associated with frailty, reduced response to the influenza vaccine and increased mortality [9, 10]. In addition, CMV has been linked to a declining immune system associated with advancing age, a concept termed ‘immunosenescence’ [11].

Furthermore, latent CMV infection has recently been linked to increased arterial stiffness, a marker of CVD risk, in patients with chronic kidney disease (CKD) [12]. CD4 + CD28null cell expansions in turn have been associated with an increased risk of CVD in patients with CKD as well as rheumatoid arthritis [13, 14]. Finally, in vitro experiments have demonstrated that CD4+ CMV-specific cells are able to target and damage the endothelium via expression of the chemokine receptors CX3C-chemokine-receptor-1 (CX3CR1) and CXCR3 that bind their respective ligands fractalkine and interferon gamma-induced protein 10 (IP-10), which are expressed on the surface of activated endothelial cells [15]. As such, considerable evidence suggests that CMV-specific CD4 + CD28null cell expansions are associated with systemic impairment and dysregulation of immune function leading to a heightened risk of infection and CVD, two of the leading causes of morbidity and mortality in AAV patients.

CMV reactivation is believed to occur in a relatively frequent basis in vivo [16–18] and is thought to result in gradual accumulation of CD4 + CD28null CMV-specific effector T cells and a reduction in CD4+ naive T cells, a phenomenon known as ‘memory inflation’ [19]. The cytokine production of CMV-specific cells may also be modulated over time correlating to loss of function. In a study of elderly CMV seropositive patients, up to 50 % of CMV-specific CD4+ T cells were unable to produce interleukin-2 (IL-2) and were capable of only interferon gamma (IFN-γ) production [20]. Associated with cytokine modulation is an increase in the expression of inhibitory receptors, cytotoxic T-lymphocyte associated protein 4 (CTLA-4), programmed cell death protein 1 (PD-1) and T-cell immunoglobulin and mucin domain containing 3 (TIM-3) leading to attenuation of virus-specific T-cell function [21]. On the other hand, several studies have described CD4 + CD28null cells as a pro-inflammatory cytotoxic subset capable of producing significant amounts of IFN-γ, granzyme B and perforin, which are implicated in endothelial damage and an increased risk of CVD as alluded to earlier [22].

Prophylactic treatment with the anti-viral agent valaciclovir can suppress CMV replication in renal transplant recipients [23] and treatment of viraemia reduces PD-1 expression and increases IL-2 production [24]. We hypothesise that repeated episodes of sub-clinical CMV replication in AAV patients drive the expansion and possible functional impairment of CD4 + CD28null CMV-specific cells with a resultant increased susceptibility to infection. We further hypothesise that inhibition of viral replication with valaciclovir will ameliorate the expansion of CD4 + CD28null cells and possibly increase the functional capacity of the immune system in patients with AAV.

We report here the study protocol for a randomised controlled open-label proof-of-concept clinical trial that aims to explore the ways in which CMV modulates the immune system in patients with AAV and investigate whether treatment with valaciclovir can ameliorate this CMV-induced adverse modulation of the immune system.

## Methods/design

This is a single-centre open-label randomised controlled trial of oral valaciclovir treatment (2 g four times a day; reduced appropriately depending on renal function) for 6 months versus no additional treatment, in CMV seropositive patients with stable AAV, followed by a 6-month follow-up period. No placebo will be used.

The primary outcome is the proportion of patients with CMV reactivation, as assessed by measurable viral load on quantitative blood and urine CMV polymerase chain reaction (PCR). The secondary outcomes are safety, as defined by adverse events sufficient to stop treatment with valaciclovir, change in the proportion of the CD4+ CMV-specific T-cell population (defined as CD4 + CD28null cells) and change in soluble markers of inflammation from baseline to 6 months (Table 1). Further tertiary and exploratory outcomes include persistence of the effect of valaciclovir on the proportion of CD4 + CD28null cells at 6 months post completion of treatment, change in the immune phenotype of CD4+ T cells and change in blood pressure and arterial stiffness parameters from baseline to 6 months (Table 1).

**Table 1:** Study endpoints

	Description	Specific measurement variable
Primary outcome		
	Proportion of patients with CMV reactivation	Quantification of viral DNA copies in blood and urine by quantitative polymerase chain reaction (qPCR)
Secondary outcomes		
	Safety	Number of adverse events and incidence of events by system organ class
		Adverse events sufficient to stop treatment with study drug
	Change in the proportion of CD4 + CD28null cells from baseline to 6 months	Proportion of CD3 + CD4 + CD28- T cells in peripheral blood
	Change in the concentration of soluble markers of inflammation from baseline to 6 months	Concentration of IL-2, tumour necrosis factor alpha (TNF-α), IFN-γ, IL-6, IL-10, IL-17 and highly sensitive C-reactive protein (CRP) in peripheral blood
Tertiary outcome		
	Persistence of valaciclovir effect on the proportion of CD4 + CD28null cells from 6 months to 12 months	Proportion of CD3 + CD4 + CD28- T cells in peripheral blood
Exploratory outcomes		
	Change in immune phenotype of CD4+ T cells from baseline to 6 months	Proportion of CD4 + CD28null cells secreting IFN-γ, IL-2, TNF-α, IL-5 and IL-10 in response to CMV lysate stimulation
		Proportion of CD4 + CD28null cells expressing the inhibitory receptors PD-1, TIM-3, CTLA-4 and lymphocyte activation gene 3 (LAG-3)
		Proportion of CD4 + CD28null cells expressing the transcription factors T box expressed in T cells (T-bet) and positive regulatory (PR) domain zinc finger protein 1 (BLIMP-1)
		Proportion of CD4 + CD28null cells expressing the chemokine receptors CX3CR1, CXCR3, C-C chemokine receptor type 5 (CCR5), CD49d and CD11b
		Proportion of CD4+ naive, central memory, effector memory and terminally differentiated effector memory populations
	Change in soluble markers of endothelial damage from baseline to 6 months	Concentration of fractalkine, IP-10, regulated on activation, normal T cell expressed and secreted (RANTES), P-selectin, E-selectin, monocyte chemoattractant protein-1 (MCP-1), soluble vascular cell adhesion molecule 1 (sVCAM-1) and soluble intracellular cell adhesion molecule 1 (sICAM-1) in peripheral blood
	Change in markers of arterial stiffness from baseline to 6 months	Measures of arterial stiffness: cfPWV, peripheral pulse pressure and central pulse pressure

Clinical trial authorisation has been obtained from the Medicines and Healthcare Products Regulatory Agency (MHRA) (EudraCT number 2012-001970-28). The trial sponsor is the University of Birmingham and the study will be run through the National Institute for Health Research (NIHR) and Wellcome Trust (WT) Clinical Research Facility (CRF) at the University Hospital Birmingham (UHB) National Health Service (NHS) Foundation Trust, which also hosts the UHB Vasculitis Clinic. The trial is funded by WT and Vasculitis UK. The trial will be coordinated by the trial management group (TMG) in conjunction with the NIHR/WT CRF according to the current guidelines for Good Clinical Practice (GCP) and ensuring protection of patients’ rights as detailed in the Declaration of Helsinki. All laboratory assays will be carried out in laboratories that fulfil the principles of Good Laboratory Practice and assays informing the primary and secondary outcomes will be fully validated prior to study commencement. The protocol was designed based on the SPIRIT guidelines (see SPIRIT checklist; Additional file 1).

### Participants

In total, 50 CMV seropositive patients with AAV in stable remission for 6 months or longer and on a maximum of two immunosuppressant agents will be recruited from the Vasculitis Clinic at UHB NHS Foundation Trust. Recruitment will take place over 2 years. Our tertiary referral vasculitis clinic has more than 200 patients under long-term follow-up. Approximately 90 % of patients are in remission at any one time and 70 % are seropositive for CMV. CMV status is checked routinely at the patient’s first attendance in the clinic. We anticipate that 125 of our patients attending the clinic will be eligible for this study. Recruitment of 40 % would achieve the target of 50 patients. This is a conservative rate given our previous experience. Patients will be approached at routine clinic visits. Patients may also be contacted via post by sending them a copy of the patient information sheet (see Additional file 2). Patients will be allowed a minimum of 24 hours to reflect on the content of the patient information sheet before written informed consent is obtained by completion and signing of the study-specific informed consent form (see Additional file 3).

A full list of study inclusion and exclusion criteria is given in Table 2. Subjects will be withdrawn from the trial if they choose not to continue or the investigators feel that continued participation in the trial is inappropriate. Subjects who withdraw from the intervention will be asked if they are prepared to continue to attend follow-up clinics. The study’s primary and main secondary outcomes are based on objective laboratory assays, therefore, minimising the risk of performance bias in this open-label design. Furthermore, treatment and medical management other than valaciclovir will be identical between the treated and control patients.

**Table 2:** Study inclusion and exclusion criteria

Inclusion criteria	
	Documented diagnosis of granulomatosis with polyangiitis (Wegener’s), microscopic polyangiitis or renal limited vasculitis according to Chapel Hill Consensus Conference Criteria
	In stable remission (no documented clinical disease activity) for at least 6 months prior to study entry
	On maintenance immunosuppression with prednisolone, mycophenolate mofetil or azathioprine alone or in combination (maximum two agents)
	Documented past evidence (any time point) of CMV infection (CMV-specific immunoglobulin G detected in peripheral blood)
	Documentation that female patients of child-bearing potential are not pregnant and are using an appropriate form of contraception
	Written informed consent for study participation
Exclusion criteria	
	Stage 5 CKD (estimated glomerular filtration rate (eGFR) <15 ml minute^-1^ 1.73 m^-2^); tests performed within 6 months of pre-baseline visit can be used for this assessment
	Other significant chronic infection (HIV, hepatitis B, hepatitis C or tuberculosis)
	B-cell depleting therapy within 12 months or T-cell depleting therapy within 6 months
	Treatment with anti-CMV therapies in the last month
	Underlying medical conditions, which in the opinion of the investigator place the patient at unacceptably high risk for participating in the study
	Inability to participate fully or appropriately in the study

### Schedule of assessments

**Fig. 1:**
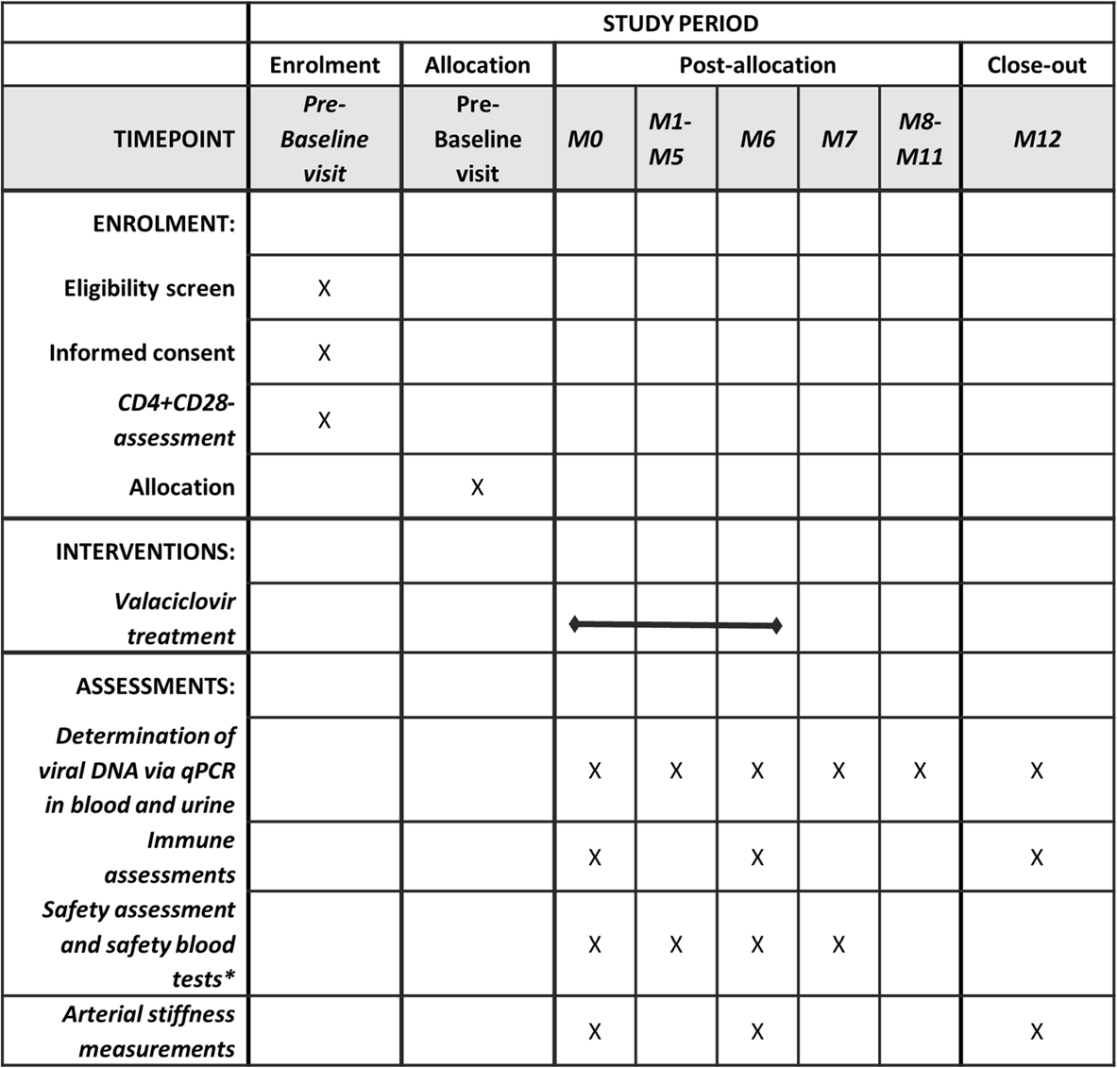
SPIRIT figure showing study’s schedule of enrolment, intervention and assessment. *M0* baseline visit, *M1* to *M12* month 1 to month 12, *qPCR* quantitative polymerase chain reaction. *Safety blood tests will be performed only on treatment group

Patients will attend for a total of 14 visits over a period of just over 12 months (Fig. 1). Evaluation of CMV reactivation by deoxyribonucleic acid (DNA) PCR of blood and urine will be done monthly. Evaluation of the tolerability of the drug and adverse events will occur monthly. Immune assessments as detailed below and in Table 1 will be performed at entry, 6 months and 12 months (Fig. 1).

After providing written informed consent, the patients will undergo an initial pre-baseline visit. During this visit, a 5-ml blood sample will be drawn, which will be used to determine the percentage of CD4 + CD28null cells. This value will be utilised in the randomisation of patients as explained below.

At the baseline, 6-month and 12-month visits, a total of 50 ml of blood will be drawn in addition to a 50-ml sample of urine. The samples will be used to determine CMV viral copies by DNA PCR in blood and urine (primary outcome), the proportion of CD4 + CD28null cells in peripheral blood by flow cytometry (secondary outcome), the concentration of soluble markers of inflammation (Table 1) in peripheral blood (secondary outcome) and immune assessments and T-cell phenotyping as detailed in the exploratory outcomes (Table 1). Excess sample will be stored appropriately. Blood pressure and arterial stiffness measurements will also be carried out at the baseline, 6-month and 12-month visits as detailed below.

During the remainder of the monthly visits, a 10-ml blood sample will be drawn in addition to a 50-ml sample of urine, which will be used to determine CMV viral copies by DNA PCR in blood and urine (primary outcome). Excess sample will again be stored appropriately. In addition, safety blood tests as defined below will be performed monthly for the duration of treatment for those patients randomised to receive the drug.

Appointments will be scheduled on monthly with an allowance of 10 days either side of the estimated due date of the monthly appointment to allow for patient convenience and flexibility. The overall treatment period (or control period) of 6 months will also be subject to the same 10-day rule to avoid excessive movement of the length of treatment.

### Study assessments

Enumeration of CMV DNA copies for the determination of the primary outcome (CMV reactivation) will be carried out by PCR of plasma and urine. This assessment will be performed by the UHB Virology Laboratory using an existing validated assay utilised for clinical samples.

Whole blood will be stained with fluorochrome conjugated monoclonal antibodies to CD3, CD4 and CD28 and analysed via flow cytometry (LSR II Flow Cytometer, DIVA Software; BD) to determine the proportion of CD4 + CD28null cells in peripheral blood. Plasma will be assessed via Luminex technology to determine the soluble markers of inflammation (Table 1). These assays will be validated for precision and reproducibility prior to commencement of the trial (see also the laboratory manual; Additional file 4).

Determination of blood pressure and arterial stiffness will be conducted using the Vicorder system (Skidmore, Bristol, UK) [25, 26]. Carotid to femoral pulse wave velocity (cfPWV) will be used to estimate arterial stiffness. The Vicorder system provides a non-invasive non-operator dependent method of obtaining cfPWV using a volume displacement method. Patients will be allowed to rest in a supine 30° head tilt position for 5 minutes prior to inflating a 100-mm-wide blood pressure cuff on the non-dominant arm to determine peripheral blood pressure. A 30-mm-wide partial cuff will be placed on the neck at the level of the carotid artery and a 100-mm-wide blood pressure cuff will be placed around the proximal thigh. The distance between the mid-clavicular point and the mid-point of the thigh cuff will be measured and entered in the Vicorder instrument as the aortic path length. The neck and thigh cuffs will then be inflated to 60 mm Hg and the Vicorder instrument will utilise the resultant oscillometric signal to extract the pulse waveforms and pulse transit time to calculate cfPWV. The mean value of three consistent recordings will be used for subsequent analysis. Inconsistent values will be re-analysed by a senior independent examiner not involved in taking the measurements to determine the validity of each measurement.

### Randomisation

Randomisation will be undertaken using the independent telephone-based randomisation system of the Primary Care Clinical Research and Trials Unit at the University of Birmingham (which is fully accredited by the NIHR as a trials unit). We will employ block randomisation by CD4 + CD28null cell percentage stratification (cut-off 40 %). The randomisation will use mixed blocks of random size (two, four or six) not known to the research team, therefore, minimising any risk of selection bias.

### Treatment

Patients randomised to the treatment arm will receive 2 g of valaciclovir orally four times a day. The dose will be reduced appropriately depending on renal function (Table 3). The study is open label and valaciclovir will be used off the shelf with no modifications to the packaging or labelling of the product. Patients will be asked to return unused tablets at each monthly visit to monitor compliance with medication. Patients randomised to the control arm will receive no additional treatment. Safety blood tests will be conducted monthly for the duration of treatment for the patients randomised to valaciclovir and these will comprise a full blood count, urea and electrolyte levels, and liver function tests. In the event of toxicity of grade 2 or less (scored using the National Cancer Institute (NCI) Common Terminology Criteria for Adverse Events version 4.0), the adverse event will be discussed with one of the investigators to determine whether drug administration should be temporarily withdrawn or the dose reduced. In the event of toxicity of grade 3 or more, the adverse event will be discussed with the principal investigator. In such a case, the expectation will be to withdraw drug administration for 1 week unless the adverse event is judged by the principal investigator to be unrelated to the study drug. Reintroduction of the drug will be based on a clinical review. The collection and reporting of data on adverse events and serious adverse events will be in accordance with EU directive 2001/20/EC and UK legislation.

**Table 3:** Dose modification of valaciclovir according to creatinine clearance

Creatinine clearance (CrCl ml/min)^a^	Valaciclovir dose
>75	2 g four times a day
51–75	1.5 g four times a day
26–50	1.5 g three times a day
10–25	1.5 g two times a day

^a^Tests performed within 6 months of the pre-baseline visit can be used for this assessment

### Sample size calculation and planned statistical analyses

In an immunocompetent elderly population, CMV reactivation occurred in 90 % at 6 months [18]. It is expected that CMV reactivation will be at least that in our immunosuppressed population. Information for dosing regimens using antiviral prophylaxis in renal transplant recipients has demonstrated over 90 % suppression of CMV reactivation. Our sample size assumes 90 % reactivation in the control limb and a conservative estimate of 50 % reactivation in the treated group. The estimated sample size is 50 patients, 25 patients in each arm, based on 80 % power at a significance level of *p* < 0.05 (two-tailed test).

All analyses will be performed using the intention-to-treat principle. Baseline covariates will be compared between the two arms to observe the balance and the success of randomisation. The primary analysis will test the hypothesis that there is no difference in the proportion of patients with CMV reactivation between those receiving antiviral prophylaxis compared with those receiving usual treatment. For the secondary outcomes, comparisons will be made between groups using absolute measures of immune function and change between data at entry and end of treatment within groups. Missing data will be dealt with by simple imputation if missing randomly. Where data are missing systematically, appropriate statistical models will be applied to avoid attrition bias. A full statistical analysis plan will be written and approved prior to data analysis.

A safety analysis will be performed on all treated patients. The number of events and incidence of adverse events by system organ class will be summarised and the relationship to the treatment noted.

The final report will follow the CONSORT 2010 guidelines and will be published in a peer-reviewed journal. Authorship will be based on the International Committee of Medical Journal Editors guidelines. Results will be communicated to study participants and patient groups through presentations at patient and carer group meetings.

### Trial management and monitoring

The trial will be coordinated by the TMG (principal investigator, co-investigators and statistical advisor) in conjunction with the NIHR/WT CRF. A trial steering committee (TSC) that will include the TMG as well as two independent consultant nephrologists not involved in the study or regular review of patients recruited in the trial will provide the overall supervision of the trial. The TSC will oversee trial progress, protocol compliance, patient safety and review of updated information. Part of the role of the TSC will be to review safety data after the first ten patients have completed treatment. As this is a small proof-of-concept study with a short follow-up, no data monitoring committee will be formed, as agreed with the sponsor. Any protocol amendments will be submitted to the sponsor and relevant regulatory bodies for approval prior to implementation and trial participants will be informed of any protocol modifications. The University of Birmingham will conduct regular audit visits in its capacity as the trial sponsor to ensure compliance with the protocol and adherence to GCP and regulations.

The integrity of data entry will be ensured using a trial-specific Data Input Quality Control standard operating procedure (Trial Master File, University of Birmingham). Samples will be anonymised and all analyses will be undertaken on anonymised datasets with study identifiers replacing personal data. All personal details will be kept on NHS secure password-protected servers within UHB NHS Trust. Anonymised data will be transferred to password-protected servers in the University of Birmingham for analysis. No data will be stored on computer hard drives. All staff have confidentiality clauses in their honorary and substantive contracts.

## Discussion

Infection and CVD represent two of the leading causes of death in patients with AAV. Expansions of CD4 + CD28null T cells in AAV that are driven by latent CMV infection have previously been shown to be associated with an increased risk of infection and mortality, whilst in other inflammatory disorders, such as CKD and rheumatoid arthritis, CD4 + CD28null cell expansions are linked to an increased risk of CVD. This randomised controlled proof-of-concept clinical trial will investigate in more detail the ways via which CMV adversely modulates the immune system in AAV and test the hypothesis that via blocking subclinical CMV reactivation with valaciclovir, it is possible to ameliorate such CMV-driven adverse modulation and improve the functional capacity of the immune system in AAV.

The results of this study will enable larger studies to be conducted to determine whether by controlling subclinical CMV reactivation in AAV, we can improve clinical endpoints, such as risk of infection, incidence of cardiovascular events and mortality. The potential impact of this study is not limited to AAV, as CD4 + CD28null cells have been linked to adverse outcomes in other inflammatory conditions and in the context of an ageing immune system.

## Trial status

As at the time of submission, the CANVAS study is in the process of patient recruitment.

### Protocol amendments

Version 1.1 was approved on 21 August 2012.

Version 1.4 was approved on 19 December 2012.

Version 1.5 was approved on 29 July 2013.

Version 1.6 was approved on 23 December 2013.

Version 1.7 was approved on 2 June 2014.

Version 2.0 was approved on 19 November 2014.

Version 2.1 was approved on 5 March 2015.

### Sponsor

The CANVAS study is sponsored by the University of Birmingham (Dr Sean Jennings, Research Governance and Ethics Manager, Research Support Group, University of Birmingham, Edgbaston, Birmingham, B15 2TT, UK). The University of Birmingham holds public liability (negligent harm) and clinical trial (negligent harm) insurance policies, which apply to this trial. The sponsor has been involved in protocol design as well as the development of the case report form and assay validation plans.

### Dissemination policy

It is anticipated that the findings of this study will be published in peer-reviewed journals and that the results will be disseminated to all study participants who wish to be informed.

## Abbreviations

AAV, ANCA-associated vasculitis; ANCA, anti-neutrophil cytoplasmic antibody; BLIMP-1, positive regulatory (PR) domain zinc finger protein 1; CCR5, C-C chemokine receptor type 5; cfPWV, carotid to femoral pulse wave velocity; CKD, chronic kidney disease; CMV, cytomegalovirus; CRF, Clinical Research Facility; CTLA-4, cytotoxic T-lymphocyte associated protein 4; CVD, cardiovascular disease; CX3CR1, CX3C-chemokine-receptor-1; DNA, deoxyribonucleic acid; eGFR, estimated glomerular filtration rate; GCP, good clinical practice; IFN-γ, interferon-gamma; IL-10, interleukin-10; IL-17, interleukin-17; IL-2, interleukin-2; IL-5, interleukin-5; IL-6, interleukin-6; IP-10, interferon gamma-induced protein 10; LAG-3, lymphocyte activation gene 3; MCP-1, monocyte chemoattractant protein-1; MHRA, Medicines and Healthcare Products Regulatory Agency; NHS, National Health Service; NIHR, National Institute for Health Research; PCR, polymerase chain reaction; PD-1, programmed cell death protein 1; qPCR, quantitative polymerase chain reaction; RANTES, regulated on activation, normal T cell expressed and secreted; sICAM-1, soluble intracellular cell adhesion molecule 1; vICAM-1, soluble vascular cell adhesion molecule 1; T-bet, T box expressed in T cells; TIM-3, T-cell immunoglobulin and mucin domain containing 3; TMG, trial management group; TNF-α, tumour necrosis factor alpha; TSC, trial steering committee; UHB, University Hospital Birmingham; WT, Wellcome Trust

## Additional files

Additional file 1: Complete SPIRIT checklist. (DOC 122 kb) Additional file 2: CANVAS Version 1.4 of the participant information sheet. (PDF 293 kb) Additional file 3: CANVAS – Consent Version 1.4 of the consent form. (PDF 139 kb) Additional file 4: CANVAS – Lab Manual Version 3.0 of the trial laboratory manual. (DOCX 181 kb)

## References

[1] Falk RJ (2011). Granulomatosis with polyangiitis (Wegener’s): an alternative name for Wegener’s granulomatosis. J Am Soc Nephrol.

[2] Jennette JC (1994). Nomenclature of systemic vasculitides. Proposal of an international consensus conference. Arthritis Rheum.

[3] Mukhtyar C (2009). EULAR recommendations for the management of primary small and medium vessel vasculitis. Ann Rheum Dis.

[4] Mukhtyar C (2008). Outcomes from studies of antineutrophil cytoplasm antibody associated vasculitis: a systematic review by the European League Against Rheumatism systemic vasculitis task force. Ann Rheum Dis.

[5] Flossmann O (2011). Long-term patient survival in ANCA-associated vasculitis. Ann Rheum Dis.

[6] Abdulahad WH, Stegeman CA, Kallenberg CG (2009). Review article: the role of CD4^+^ T cells in ANCA-associated systemic vasculitis. Nephrology.

[7] Morgan MD (2011). CD4 + CD28- T cell expansion in granulomatosis with polyangiitis (Wegener’s) is driven by latent cytomegalovirus infection and is associated with an increased risk of infection and mortality. Arthritis Rheum.

[8] Trzonkowski P (2010). Immunosenescence increases the rate of acceptance of kidney allotransplants in elderly recipients through exhaustion of CD4+ T-cells. Mech Ageing Dev.

[9] Vescovini R (2010). Intense antiextracellular adaptive immune response to human cytomegalovirus in very old subjects with impaired health and cognitive and functional status. J Immunol.

[10] Derhovanessian E (2014). Latent infection with cytomegalovirus is associated with poor memory CD4 responses to influenza A core proteins in the elderly. J Immunol.

[11] Trzonkowski P (2003). Association between cytomegalovirus infection, enhanced proinflammatory response and low level of anti-hemagglutinins during the anti-influenza vaccination – an impact of immunosenescence. Vaccine.

[12] Walldorf J, Lubbert C (2013). Pitfalls in diagnosis and therapy for CMV colitis in a 30-year-old HIV-positive patient. Z Gastroenterol.

[13] Gerli R (2004). CD4 + CD28- T lymphocytes contribute to early atherosclerotic damage in rheumatoid arthritis patients. Circulation.

[14] Yadav AK, Lal A, Jha V (2012). Cytotoxic CD4^+^CD28^null^ T lymphocytes, systemic inflammation and atherosclerotic risk in patients with chronic kidney disease. Nephron Clin Pract.

[15] van de Berg PJ (2012). Cytomegalovirus-induced effector T cells cause endothelial cell damage. Clin Vaccine Immunol.

[16] Docke WD (2003). Subclinical activation of latent cytomegalovirus (CMV) infection and anti-CMV immune response in patients with atopic dermatitis. Br J Dermatol.

[17] Ling PD (2003). The dynamics of herpesvirus and polyomavirus reactivation and shedding in healthy adults: a 14-month longitudinal study. J Infect Dis.

[18] Stowe RP (2007). Chronic herpesvirus reactivation occurs in aging. Exp Gerontol.

[19] Pourgheysari B (2007). The cytomegalovirus-specific CD4+ T-cell response expands with age and markedly alters the CD4+ T-cell repertoire. J Virol.

[20] Kannanganat S (2007). Multiple-cytokine-producing antiviral CD4 T cells are functionally superior to single-cytokine-producing cells. J Virol.

[21] Yi JS, Cox MA, Zajac AJ (2010). T-cell exhaustion: characteristics, causes and conversion. Immunology.

[22] Dumitriu IE. The life (and death) of CD4 CD28 T cells in inflammatory diseases. Immunology, 2015;146(2):185–93. 10.1111/imm.12506 PMC4582960 26190355

[23] Reischig T (2008). Valacyclovir prophylaxis versus preemptive valganciclovir therapy to prevent cytomegalovirus disease after renal transplantation. Am J Transplant.

[24] Sester U (2008). PD-1 expression and IL-2 loss of cytomegalovirus-specific T cells correlates with viremia and reversible functional anergy. Am J Transplant.

[25] Hickson SS (2009). Validity and repeatability of the Vicorder apparatus: a comparison with the SphygmoCor device. Hypertens Res.

[26] Ng KP (2014). Allopurinol is an independent determinant of improved arterial stiffness in chronic kidney disease: a cross-sectional study. PLoS One.

