# Supplementary material for: Valaciclovir to prevent Cytomegalovirus mediated adverse modulation of the immune system in ANCA-associated vasculitis (CANVAS): study protocol for a randomised controlled trial
Source: Trials. 2016 Jul 22;17:338. doi: 10.1186/s13063-016-1482-2 (PMC4957324; doi:10.1186/s13063-016-1482-2)
Supplement: Additional file 2: — CANVAS Version 1.4 of the participant information sheet. (PDF 293 kb) [file 13063_2016_1482_MOESM2_ESM.pdf]

C A N V A S

CMV modulation of the immune system in **AN**CA-associated **VAS**culitis

Patient (Active) Information Sheet

We would like to invite you to take part in a research study. Before you decide whether or not you wish to take part, it is important for you to understand why the research is being done and what it will involve. Please take time to read the following information carefully and discuss it with others if you wish. It explains why we are undertaking this study, what your involvement would be and hopefully answers any questions that you may have at this stage.

What is the purpose of the study?
Cytomegalovirus (CMV) is a common virus that tends not to cause symptoms in healthy individuals. It is present in a large proportion of the population such that by middle age over half the population have had CMV infection. Research has shown that following initial infection the virus remains in the body for life and undergoes periods of reactivation. The immune system normally keeps the virus under control. However this means that a large proportion of T-cells, a part of the immune system important for fighting infection, are 'committed' to controlling CMV. Eventually some of these committed T-cells become “exhausted” and do not appear to work well. The presence of large numbers of “exhausted” immune system cells in the body may impair the ability to control CMV and fight other infections. In most studies this only appears to be a problem very late in life.

However in previous studies in Birmingham we have shown that vasculitis patients who have CMV infection and a lot of “exhausted” cells seem to be more prone to infections. This is important as the treatment for vasculitis also suppresses immune system activity and increases the risk of infection. In addition, CMV infection has recently been linked to increased stiffness of the blood vessels. Increased stiffness of the blood vessels is a risk factor for heart disease and stroke.

There are two main parts to this study.
Firstly we want to further investigate the nature of CMV committed T-cells and “exhausted” cells in patients and healthy people who have CMV infection.
Secondly we want to find out if using medication (Valaciclovir) that blocks CMV activity in the body may allow the immune system and the “exhausted” cells to recover which should then improve the body’s ability to fight other infections as well.

Why have I been invited?
You have been invited to participate because you have stable vasculitis disease and are positive for previous CMV infection.

Do I have to take part?
It is up to you to decide. We will describe the study and go through this information sheet, which we will then give to you. If you decide to participate we will ask you to sign a consent form to show you have agreed to take part. You are free to withdraw at any time, without giving a reason. This will not affect the standard of care you receive.

What will happen to me if I take part?
If you agree to take part we will ask you to provide an initial 5ml blood sample in order to determine the percentage of a particular subset of T-cells linked to CMV. We will then ask you to provide a 50ml (3 tablespoons) blood and urine sample so that we can measure the number of CMV 'committed' T-cells in your blood and confirm that you have had previous CMV infection. We would also want to store some of the cells frozen in liquid nitrogen for use at a later date, as we cannot run all the tests on the day you come to clinic. Additionally, we would like to save some of the blood and urine sample to measure other inflammatory chemicals and proteins and response to other viruses.

Following initial assessment you will be randomly assigned (like tossing a coin) to either continue with your regular vasculitis treatment OR receive additional treatment with an antiviral drug called valaciclovir. Treatment will be in the form of tablet therapy four times a day. There is no need for hospital admission in order to commence treatment. The medication will need to be taken every day for the prescribed length of time (6 months). We will make an appointment for you to be assessed by the research team during a brief hospital visit on a monthly basis for 12 months following commencement of your participation in the study. At each monthly visit we will also ask your permission to collect a further 10ml blood and urine sample to assess how active CMV is in your body as well as a 50ml blood and urine sample at 6 months and 12 months. If you have been assigned to take the antiviral drug we will also check your well-being at each monthly visit.
We would also like to measure your blood pressure as well as the stiffness of your blood vessels at the initial visit, 6 months and 12 months. The equipment used is very similar to a blood pressure machine and the cuffs can be placed over clothing. There are no risks to this procedure, although the inflated cuff can sometimes exert discomfort on the arm.

What are the possible disadvantages and risks of taking part?
All drugs have side effects, and no drug is without risk. However, the drug used in this study is a licensed medication and the research team will be able to adjust the dose should you suffer any side effects.

Common side effects experienced by between 1 in 10 and 1 in 100 patients include headache and nausea. Uncommon side effects experienced by between 1 in 100 and 1 in 1000 patients are shortness of breath and rashes or sensitivity to light. Rare side effects experienced by between 1 in 1,000 and 1 in 10,000 patients include dizziness, confusion, hallucinations, drowsiness, itching, kidney problems, stomach pains, vomiting and diarrhoea. Finally, very rare side effects experienced by less than 1 in 10,000 patients may include allergic reactions, anaemia, inflammation of the liver, acute kidney failure, agitation, tremors and psychosis.

What are the possible benefits of taking part?
There is no immediate benefit in participating in this study. By improving our understanding of the way CMV affects the immune system in vasculitis and determining whether treatment of CMV ameliorates these effects we may be able to improve therapy for vasculitis patients and other patient groups in the future.

What will happen if I don't want to carry on with the study?
You are free to withdraw from the study at any time and this will not affect your care. You can either withdraw completely or choose to keep in contact with us to let us know your progress. Information collected earlier in the study may still be used.

What if there is a problem?
If you have a concern about any aspect of this study, you should ask to speak to the researcher (see contact details) who will do their best to answer your questions. If you remain unhappy and wish to complain formally, you can do this through the NHS Complaints Procedure (or a private institution). Details can be obtained from the hospital.

Will my taking part in the study be kept confidential?
All information collected about you during the course of the research will be kept strictly confidential, and any information about you that leaves the hospital/clinical laboratory will have your name and address removed so that you cannot be recognised.

Involvement of the General Practitioner/Family doctor (GP)
Your GP will be informed of your participation in this study in order to monitor for any side-effects associated with the study medication.

What will happen to any samples I give?
Blood samples that you give will be sent to the University Hospital Birmingham Virology Laboratory and our research laboratories (Wellcome Trust Clinical Research Facility (WTCRF) and University of Birmingham Laboratories). Some tests will be done on your blood samples shortly after the samples have been taken. The remaining sample will then be placed in a freezer (WTCRF and University of Birmingham), as other tests will be done some time later. The cells will not be used for financial profit. We would like your permission to retain any cells left at the end of the project for use in future projects (subject to approval by the Research Ethics Committee).

Will any genetic tests be done?
The only genetic test performed will be an assessment of your HLA type (a marker on the surface of white blood cells) as this is essential in carrying out part of the T-cell analysis. This will not produce any findings of clinical significance for yourself or any of your relatives.

What will happen to the results of the research study?
The overall broad scientific results of the study will be published in medical/scientific journals and the publications made available to all participants. You will not be identified in any report or publication.

Who is organising and funding the research?
The study is being organised by the University of Birmingham. The research is being funded by the

Wellcome Trust and the Vasculitis UK (formerly Stuart Strange Trust) patient support group.

Who has reviewed the study?

All research in the NHS is reviewed by an independent group of people, called a Research Ethics Committee to protect your safety, rights, wellbeing and dignity. This study has been reviewed by the NRES Committee York and Humber - Leeds West Research Ethics Committee and has been given a favourable opinion. The scientific rigour of this study has been reviewed by the Wellcome Trust who have agreed to fund this research. The study has also been reviewed by the trustees of Vasculitis UK who think this is an important study for vasculitis patients.

Further information and contact details

For further information, please contact:

Professor Lorraine Harper, Professor Paul Moss, Dr Matthew Morgan or Dr Dimitrios Chanouzas via the Wellcome Trust Clinical Research Facility at the University Hospitals Birmingham NHS Foundation Trust. Telephone: 0121 371 3170.

Thank you for taking time to consider participating in this study.

Supported by
wellcome trust

VASCULITIS UK
