# Supplementary material for: Valaciclovir to prevent Cytomegalovirus mediated adverse modulation of the immune system in ANCA-associated vasculitis (CANVAS): study protocol for a randomised controlled trial
Source: Trials. 2016 Jul 22;17:338. doi: 10.1186/s13063-016-1482-2 (PMC4957324; doi:10.1186/s13063-016-1482-2)
Supplement: Additional file 3: — CANVAS – Consent Version 1.4 of the consent form. (PDF 139 kb) [file 13063_2016_1482_MOESM3_ESM.pdf]

Name:

Date of Birth: Hospital number:

Subject Identification Number for this study:

Patient active consent form

Title of Project: **C**MV modulation of the immune system in **AN**CA-associated **VAS**culitis (CANVAS)
Name of Researcher: Professor Lorraine Harper

Please initial boxes

1. I confirm that I have read and understood the Patient Information Sheet (Version 1.4) for the above study. I have had the opportunity to consider the information, ask questions and have had these answered satisfactorily. ☐

2. I understand that my participation is voluntary and that I am free to withdraw at any time without giving any reason, without my medical care or legal rights being affected. ☐

3. I understand that relevant sections of my medical notes and data collected during the study, may be looked at by individuals from the University of Birmingham, from regulatory authorities or from the NHS Trust, where it is relevant to my taking part in this research. I give permission for these individuals to have access to my records. ☐

4. I understand that my GP will be informed of my participation in this study and may be contacted to provide information about my progress, in confidence, to the organisers. ☐

5. I understand participation is a 12-month commitment. I agree to attend clinics and donate blood and urine samples for study as detailed in the information sheet. ☐

6. I agree that my samples may be stored within the University of Birmingham for future research, providing ethical approval for these additional studies has first been obtained. ☐

7. I also understand that data collected about me for this study is covered under the Data Protection Act 1998 and stored electronically in a secure encoded format. ☐

8. I agree to take part in the above study. ☐

Name of Patient	Date	Signature
Name of Person taking consent	Date	Signature

When completed, 1 for patient; 1 for researcher site file; 1 (original) to be kept in medical notes

Supported by wellcome trust

VASCULITIS UK
