# Supplementary material for: Valaciclovir to prevent Cytomegalovirus mediated adverse modulation of the immune system in ANCA-associated vasculitis (CANVAS): study protocol for a randomised controlled trial
Source: Trials. 2016 Jul 22;17:338. doi: 10.1186/s13063-016-1482-2 (PMC4957324; doi:10.1186/s13063-016-1482-2)
Supplement: Additional file 4: — CANVAS – Lab Manual Version 3.0 of the trial laboratory manual. (DOCX 181 kb) [file 13063_2016_1482_MOESM4_ESM.docx]

**LABORATORY MANUAL**

Version 3.0

15.06.2015

| **Author:** | **Dr Dimitrios Chanouzas** |
| --- | --- |
| **Role:** | **Analytical Project Manager** |
| **Signature:** |  |

**Table of Contents**

1. **Contents Page 2**
2. **Key Names & Contact Details Page 3**
3. **Sample Pathway Page 4**
4. **Whole blood / positive control surface staining Work Instruction Page 6**
5. **Whole blood / positive control surface staining Assay Record Page 8**
6. **Processing of plasma by CRF Lab Page 9**
7. **Blood sample receipt and processing record Page 10**
8. **Processing of 6ml blood sample at month 1-5 and month 7-11**

**visits by CRF Lab Page 11**

1. **Processing of urine samples by CRF Lab Page 12**

**2. Key Names and Contact Details**

**TRIAL MANAGEMENT GROUP**

**Chief Investigator**

**Professor Lorraine Harper**

Professor of Nephrology

School of Immunity and Infection

Centre for Translational Inflammation Research

University of Birmingham Research Laboratories

Queen Elizabeth Hospital Birmingham

Mindelsohn Way, Edgbaston

Birmingham, B15 2WB

Telephone: +44 (0)121 371 3238 E-mail: [l.harper@bham.ac.uk](mailto:l.harper@bham.ac.uk)

**Co-Investigators**

**Professor Paul Moss**

Professor of Haematology

School of Cancer Sciences

University of Birmingham

Edgbaston

Birmingham, B15 2TT

Telephone: +44 (0)121 414 2824 E-mail: [p.moss@bham.ac.uk](mailto:p.moss@bham.ac.uk)

**Dr Matthew Morgan**

Senior Lecturer (Clinical) in Renal Medicine

School of Immunity and Infection

Centre for Translational Inflammation Research

University of Birmingham Research Laboratories

Queen Elizabeth Hospital Birmingham

Mindelsohn Way, Edgbaston

Birmingham, B15 2WB

Telephone: +44 (0)121 371 3223 E-mail: [m.d.morgan@bham.ac.uk](mailto:m.d.morgan@bham.ac.uk)

**Dr Dimitrios Chanouzas**

Wellcome Trust Research Fellow

School of Immunity and Infection

Centre for Translational Inflammation Research

University of Birmingham Research Laboratories

Queen Elizabeth Hospital Birmingham

Mindelsohn Way, Edgbaston

Birmingham, B15 2WB

Telephone: +44 (0) 7971 402309 E-mail: [dgchanouzas@doctors.org.uk](mailto:dgchanouzas@doctors.org.uk)

1. **Sample Pathway**

Following informed consent patient visits will take place at the NIHR / Wellcome Trust Clinical Research Facility (CRF) – see also CANVAS Sample Collection Work Instruction (APPENDIX A)

1. **5ml** **Blood** Sample Collection Prior to Baseline **(Pre-Baseline)**
   1. Collected in Green Lithium Heparin Tube
      1. Research Fellow to pick up from CRF
      2. Sample used to determine CD3+CD4+CD28- % prior to randomisation
         1. Process sample according to Work Instruction detailed in Section 4 of this manual
         2. Keep assay record using form enclosed in Section 5 of this manual
         3. No sample storage necessary

1. **50ml** **Blood** Sample Collection **at** **baseline (0 months), 6 months and 12 months**
   1. **2** x 4ml EDTA (Purple) Tubes
      1. 1 x 4ml EDTA Tube sent to QE Virology Lab by CRF research nurse – Primary Outcome Determination (CMV PCR)
      2. 1 x 4mL EDTA Tube given to CRF Lab by CRF research nurse
         1. Processed by CRF Lab according to agreed protocol detailed in Section 6 of this manual
            1. Sample will be used to determine plasma level of inflammatory mediators / cytokines – Secondary Outcome Determination
            2. Plasma samples will be stored at CRF freezers as per local procedures – sample inventory and freezer temperature logs kept locally
   2. **7** x 6ml Lithium Heparin (Green) Tubes
      1. Research Fellow to pick up from CRF
         1. Process sample and keep record according to Work Instruction detailed in Section 7 of this manual
            1. Plasma and PBMC pellet samples will be stored at Schools of Cancer Sciences and Immunity and Infection freezers – sample inventory kept within CANVAS Laboratory Folder; Freezer temperature logs kept locally by Schools of Cancer Sciences and Immunity and Infection
            2. PBMC samples will be stored at School of Immunity and Infection, Institute of Biomedical Research (IBR) liquid nitrogen tanks – sample inventory kept within CANVAS Laboratory Folder
         2. Aliquot of whole blood used to determine CD3+CD4+CD28-% - Secondary Outcomes Determination (rest of sample to be used for exploratory research outcomes and stored as above)
            1. Process sample according to Work Instruction detailed in Section 4 of this manual
            2. Keep assay record using form enclosed in Section 5 of this manual
2. **10ml** **Blood** Sample Collection **at** **monthly visits (except 0, 6 and 12 month visits)**
   1. **1** x 4ml EDTA (Purple) Tube sent to QE Virology Lab by CRF research nurse – Primary Outcome Determination (CMV PCR)
   2. **1** x 6ml Lithium Heparin (Green) Tube given to CRF Lab by CRF research nurse
      1. Processed by CRF Lab according to agreed protocol detailed in Section 8 of this manual
         1. Sample used for exploratory research outcomes
         2. PBMC samples will be stored at School of Immunity and Infection, IBR liquid nitrogen tanks – sample inventory kept within CANVAS Laboratory Folder
3. **Urine** Sample Collection **at** **All Time Points (Except Pre-Baseline)**
   1. **5-10 ml** sent to QE Virology Lab by CRF research nurse – Primary Outcome Determination (CMV PCR)
   2. Rest of Urine Sample given to CRF Lab by CRF research nurse
      1. Sample processed according to CRF laboratory standard protocol outlined in Section 9 of this manual
         1. Sample used for exploratory research outcomes
         2. Urine samples will be stored at CRF freezers as per local procedures – sample inventory and freezer temperature logs kept locally
4. **Whole blood / positive control surface staining Work Instruction**

# Purpose:

This Work Instruction describes the protocol for the surface staining of lymphocytes in whole blood / CD4 positive controls.

# REAGENTS: Product No

- Cytofix CD4 Normal Positive Control Cytomark / Caltag MedSystems CF04-N
- Anti-Human CD3 Brilliant Violet 650 Biolegend 317323
- Anti-Human CD4 Brilliant Violet 605 Biolegend 317438
- Anti-Human CD28 eFluor 450 Ebioscience 48-0289-42
- Anti-Human CD4 eFluor 450 Ebioscience 48-0047-42
- Mouse IgG2a K Isotype Control BV 650 Biolegend 400265
- Mouse IgG2b K Isotype Control BV 605 Biolegend 400350
- Mouse IgG1 K Isotype Control eFluor450 Ebioscience 48-4714-82
- 1x RBC Lysis Buffer Ebioscience 00-4333-57
- MACS Buffer School of Cancer Sciences

# Materials & methods:

- Disposable Gilson Pipette Tips
- 5 ml FACS tubes
- Pipette-Boy
- Full Set of Calibrated Pipettes
- Microbiological Safety Cabinet

# Work Instruction

1. Label 5ml FACS tubes.

2. Prepare master mix of CD3 (2uL / test), CD4 (2uL / test), and CD28 (3uL / test) antibodies on the day of intended use. Make up to total volume of 20uL per test with MACS buffer.

3. Aliquot 50uL of Cytofix CD4 Normal positive control or 100uL of whole blood into each tube.

4. Add the appropriate volume of CD3 CD4 CD28 master mix in each experimental tube and pulse vortex to mix.

5. *Also prepare 3 single stain compensation tubes and an unstained tube each containing 100uL of whole blood.*

*6. For each single stain compensation tube add the appropriate volume of CD3, CD4 or CD28 antibody (2uL / test for BV605 and BV650 and 3uL / test for eFluor450). Do not add antibody to the unstained tube.*

*7. Also prepare 1 Fluorescence Minus One (FMO) tube containing 100uL of whole blood.*

*8. For the FMO add the appropriate volume of antibody / isotype control as below.*

| **CD28 FMO** |
| --- |
| 2uL BV 650 CD3 |
| 2uL BV 605 CD4 |
| 0.5uL eFluor 450 Isotype |

9. Incubate for 30 minutes at 4 degrees Celsius in the dark.

10. Add 2ml of 1x RBC Lysis Buffer and pulse vortex.

11. Incubate for 15 minutes at room temperature in the dark.

12. Without washing centrifuge cells at 1600 rpm for 5 minutes, discard supernatant and gently resuspend cells.

13. Add 2ml MACS buffer per tube and centrifuge again as above.

14. Acquire the data on an LSR II Flow Cytometer and analyse the data using FACS DIVA Software.

1. **Whole blood / positive control surface staining Assay Record**

**Whole Blood / Positive Control Surface Staining Assay Record**

**Date of Experiment _______________**

**Type of Experiment (i.e. validation or clinical trial sample) _______________**

**Unique Patient ID (if clinical trial sample) _______________**

**Time point (i.e. 0, 6, 12 months if clinical trial sample) _______________**

**Length of time from drawing blood sample to processing _______________**

**Reagents used:**

CD3 Brilliant Violet 650 Monoclonal Antibody Lot______________ Expiry Date________

CD4 Brilliant Violet 605 Monoclonal Antibody Lot______________ Expiry Date________

CD28 eFluor 450 Monoclonal Antibody Lot______________ Expiry Date________

CD4 eFluor 450 Monoclonal Antibody Lot______________ Expiry Date________

Brilliant Violet 650 Isotype Control Lot______________ Expiry Date________

Brilliant Violet 605 Isotype Control Lot______________ Expiry Date________

eFluor 450 Isotype Control Lot______________ Expiry Date________

Cytofix CD4 Normal Positive Control Lot______________ Expiry Date________

1x RBC Lysis Buffer Solution Lot______________ Expiry Date________

**Pipettes used:**

_________________________________ Calibration due on _____________________

_________________________________ Calibration due on _____________________

_________________________________ Calibration due on _____________________

_________________________________ Calibration due on _____________________

_________________________________ Calibration due on _____________________

1. **Processing of plasma by WTCRF Lab**

**Protocol for Processing of Plasma Samples – Author Dr Julie Williams (Former WTCRF Lab Manager) (edited by Dr D Chanouzas)**

| **Receive** | **Processing** | **Storage** |
| --- | --- | --- |
| 4ml **Purple EDTA** for plasma | Spin within 30min  10min 3000rpm at 4 degrees Celsius  Divide between 4 tubes | Store at -80 degrees Celsius  Samples will be stored at WTCRF facilities until the end of the study at which point they will be taken to University of Birmingham Laboratories for batch assaying / analysis. |

1. **Blood Sample Receipt and Processing Record – University of Birmingham – CANVAS Trial**

*Unique identifier _______________*

*Date _______________ Time point in study ________________*

*Sample Received from WTCRF _______________ Sample Condition ________________*

*Length of time from drawing blood sample to processing ______________*

*Received and Processed by _______________*

**Remember to label all aliquots with ID, time-point, date, volume, type of material and concentration if applicable**

**FIRST USE SMALL ALIQUOT OF WHOLE BLOOD TO RUN SURFACE STAINING FOR 2^o^ OUTCOME (Section 4, CANVAS Lab Manual)**

Spin 1 green tube at 1600rpm for 10 minutes and aliquot plasma in 4 parts of 250uL

Location in -80 _______________

Process blood to acquire PBMC

PBMC Count _______________

PBMC used for fresh functional experiments ________________

Process 1x10^6^ cells (x2) if enough sample for DNA

Spin in micro-centrifuge at 4000rpm for 5 minutes

Carefully aspirate supernatant and store at -80

Location in -80 ______________

Freeze remainder of PBMC; Freeze as many cells as possible but ensure at least 2 tubes of 5x10^6^ cells each in 0.5ml are frozen to enable analysis of 1x10^6^ cells x 2.

Number of cells frozen ___________

Number of cryovials, volume and concentration ____________

Location in -80 __________ (Transfer to liquid nitrogen within 7 days)

Location in liquid nitrogen ___________

1. **Processing of 6ml blood sample at month 1-5 and month 7-11**

**visits by WTCRF Lab – Work Instruction**

1. Centrifuge within 2 hours at 584 g for 10 minutes.
2. Carefully aspirate 250uL of plasma x 4 and aliquot in 4 tubes.
3. Label tubes with Sample Type, Sample Volume, Unique Patient ID, Patient Initials, Study Visit, Date and Study Name (CANVAS) and store at -80 degrees Celsius.
4. Invert green lithium heparin tube a few times to re-suspend blood sample again.
5. Dilute blood sample 1:1 with RPMI pre-warmed to 37 degrees Celsius: Pour contents of blood sample tube in a 50ml tube, then add 5ml RPMI into empty blood sample tube and mix with remaining blood. Pour RPMI from the blood sample tube into the 50ml tube containing the blood sample.
6. Add 15ml Ficoll Paque into a fresh 50ml tube.
7. Carefully layer the diluted blood sample over the Ficoll layer in order to get two distinct layers.
8. Spin at **room** temperature at 584 g for 30 minutes with the **brake off**.
9. Carefully harvest the buffy coat using a Pasteur pipette into a fresh 50ml tube.
10. Top up to 40ml with RPMI and spin at 912 g for 7 minutes.
11. Carefully discard supernatant and re-suspend the PBMC pellet.
12. Top up to 40ml with RPMI and spin at 584 g for 7 minutes.
13. Carefully discard supernatant and re-suspend the PBMC pellet.
14. Add 10ml of RPMI and take 10uL aliquot to count cells using a haemocytometer or automated cell counter.
15. Centrifuge sample at 584 g for 7 minutes.
16. Carefully discard supernatant and re-suspend the cells at 20 x 10^6^ cells / ml in Freezing Solution A.
17. Add an equal volume of freezing solution B to the cells (dropwise fashion) with gentle agitation of the tube.
18. Transfer to cryovials – label cryovials with Sample type, Sample Volume, Unique Patient ID, Patient Initials, Study Visit, Date and Study Name (CANVAS).
19. Place cryovials in Mr Frosty for minimum of 4 hours at -80 degrees Celsius.
20. Transfer to liquid nitrogen within 7 days.

Freezing Solution A: 60% Fetal calf serum (FCS) and 40% RPMI

Freezing Solution B: 20% DMSO and 80% FCS

1. **Processing of urine samples by WTCRF Lab**

- **Mix well**
- **Split into two aliquots**
- **Label Aliquot A as Unspun along with the other agreed identifiers and store at -80 degrees Celsius**
- **Label Aliquot B as Spun along with the other agreed identifiers; Spin at 1000 rpm, at room temperature for 1 minute; Store at -80 degrees Celsius**
- **Samples will be stored at WTCRF facilities until the end of the study at which point they will be taken to University of Birmingham Laboratories for assaying / analysis.**

APPENDIX A

**Sample Collection WORK INSTRUCTION: CANVAS**

**WORK INSTRUCTION for Sample Collection**

Version 1.2

15.06.2015

Key Contact Details

Principal Investigator

Professor Lorraine harper [l.harper@bham.ac.uk](mailto:l.harper@bham.ac.uk)

Co-Investigators

Dr Matthew Morgan [m.d.morgan@bham.ac.uk](mailto:m.d.morgan@bham.ac.uk)

Professor Paul Moss [p.moss@bham.ac.uk](mailto:p.moss@bham.ac.uk)

Research Fellow

Dr Dimitrios Chanouzas [dgchanouzas@doctors.org.uk](mailto:dgchanouzas@doctors.org.uk)

07971402309

Research Nurses

Linda Coughlan [linda.coughlan@uhb.nhs.uk](mailto:linda.coughlan@uhb.nhs.uk)

Annabel Grinbergs [annabel.grinbergs@uhb.nhs.uk](mailto:annabel.grinbergs@uhb.nhs.uk)

WTCRF Laboratory

Mariea Parvaz [mariea.parvaz@uhb.nhs.uk](mailto:mariea.parvaz@uhb.nhs.uk)

Clinical Trial Participants (Intended number = 50)

Following informed consent patient visits will take place at the Wellcome Trust Clinical Research Facility (WTCRF)

1. **5ml** **Blood** Sample Collection Prior to Baseline **(Pre-Baseline)**
   1. Collect in Green Lithium Heparin Tube - Label with Unique Patient ID, Patient Initials, Study Visit, Date and Study Name (CANVAS)
      1. Contact Research Fellow (07971402309) to collect within 30 minutes of blood being drawn
2. **50ml** **Blood** Sample Collection **at** **baseline (0 months), 6 months and 12 months**
   1. **2** x 4ml EDTA (Purple) Tubes
      1. Send 1 x 4ml EDTA Tube to QE Virology – Ensure Fully Labelled and P386 Sticker Affixed
      2. Send 1 x 4mL EDTA Tube to WTCRF Lab – Label with Unique Patient ID, Patient Initials, Study Visit, Date and Study Name (CANVAS)
         1. Spin down according to agreed protocol (Appendix A) and aliquot plasma to 4 parts (Label as above) – freeze at -80 degrees Celsius
   2. **7** x 6ml Lithium Heparin (Green) Tubes – Label with Unique Patient ID, Patient Initials, Study Visit, Date and Study Name (CANVAS)
      1. Contact Research Fellow (07971402309) to collect Green Tubes within 30 minutes of blood being drawn
3. **10ml** **Blood** Sample Collection **at** **monthly visits (except 0, 6 and 12 month visits)**
   1. **1** x 4ml EDTA (Purple) Tube
      1. Send to QE Virology – Ensure Fully Labelled and P386 Sticker Affixed
   2. **1** x 6ml Lithium Heparin (Green) Tube – Label with Unique Patient ID, Patient Initials, Study Visit, Date and Study Name (CANVAS)
      1. Send to WTCRF Lab
         1. Process according to agreed protocol (Appendix C)
4. **Urine** Sample Collection **at** **All Time Points (Except Pre-Baseline)**
   1. Send **5-10 ml** to QE Virology – Ensure Fully Labelled and P386 Sticker Affixed
   2. Rest of Urine Sample to be sent to WTCRF Lab – Label with Unique Patient ID, Patient Initials, Study Visit, Date and Study Name (CANVAS)
      1. Spin Down according to WTCRF laboratory standard protocol (Appendix B) and Aliquot into 2 parts (Label as Above)
         1. Freeze at -80 Degrees Celsius

**Any problems or questions please contact Linda Coughlan, Annabel Grinbergs or Dr Dimitrios Chanouzas**

**APPENDIX A**

**Protocol for Processing of Plasma Samples – Author Dr Julie Williams (Former WTCRF Lab Manager) (edited by Dr D Chanouzas)**

| **Receive** | **Processing** | **Storage** |
| --- | --- | --- |
| 4ml **Purple EDTA** for plasma | Spin within 30min  10min 3000rpm at 4 degrees Celsius  Divide between 4 tubes | Store at -80 degrees Celsius  Samples will be stored at WTCRF facilities until the end of the study at which point they will be taken to University of Birmingham Laboratories for batch assaying / analysis. |

**APPENDIX B**

**Protocol for Processing of Urine Samples**

- **Mix well**
- **Split into two aliquots**
- **Label Aliquot A as Unspun along with the other agreed identifiers and store at -80 degrees Celsius**
- **Label Aliquot B as Spun along with the other agreed identifiers; Spin at 1000 rpm, at room temperature for 1 minute; Store at -80 degrees Celsius**
- **Samples will be stored at WTCRF facilities until the end of the study at which point they will be taken to University of Birmingham Laboratories for assaying / analysis.**

**APPENDIX C**

**Work instruction for processing of 6ml blood sample collected in GREEN LITHIUM HEPARIN TUBE at month 1-5 and month 7-11 study visits**

1. Centrifuge within 2 hours at 584 g for 10 minutes.

2. Carefully aspirate 250uL of plasma x 4 and aliquot in 4 tubes.

3. Label tubes with Sample Type, Sample Volume, Unique Patient ID, Patient Initials, Study Visit, Date and Study Name (CANVAS) and store at -80 degrees Celsius.

4. Invert green lithium heparin tube a few times to re-suspend blood sample again.

5. Dilute blood sample 1:1 with RPMI pre-warmed to 37 degrees Celsius: Pour contents of blood sample tube in a 50ml tube, then add 5ml RPMI into empty blood sample tube and mix with remaining blood. Pour RPMI from the blood sample tube into the 50ml tube containing the blood sample.

6. Add 15ml Ficoll Paque into a fresh 50ml tube.

7. Carefully layer the diluted blood sample over the Ficoll layer in order to get two distinct layers.

8. Spin at **room** temperature at 584 g for 30 minutes with the **brake off**.

9. Carefully harvest the buffy coat using a Pasteur pipette into a fresh 50ml tube.

10. Top up to 40ml with RPMI and spin at 912 g for 7 minutes.

11. Carefully discard supernatant and re-suspend the PBMC pellet.

12. Top up to 40ml with RPMI and spin at 584 g for 7 minutes.

13. Carefully discard supernatant and re-suspend the PBMC pellet.

14. Add 10ml of RPMI and take 10uL aliquot to count cells using a haemocytometer or automated cell counter.

15. Centrifuge sample at 584 g for 7 minutes.

16. Carefully discard supernatant and re-suspend the cells at 20 x 10^6^ cells / ml in Freezing Solution A.

17. Add an equal volume of freezing solution B to the cells (dropwise fashion) with gentle agitation of the tube.

18. Transfer to cryovials – label cryovials with Sample type, Sample Volume, Unique Patient ID, Patient Initials, Study Visit, Date and Study Name (CANVAS).

19. Place cryovials in Mr Frosty for minimum of 4 hours at -80 degrees Celsius.

20. Transfer to liquid nitrogen within 7 days.

Freezing Solution A: 60% Fetal calf serum (FCS) and 40% RPMI

Freezing Solution B: 20% DMSO and 80% FCS
